# Effect of Single Loading Time to the Cyclic Ablation of C/C-SiC-ZrC Composite

**DOI:** 10.3390/ma15197027

**Published:** 2022-10-10

**Authors:** Wei Feng, Xinyu Wang, Yunlong Tian, Lei Liu, Boyan Li

**Affiliations:** School of Materials and Chemical Engineering, Xi’an Technological University, Xi’an 710021, China

**Keywords:** cyclic ablation, single loading time, C/C composite, SiC-ZrC, plasma

## Abstract

To understand the influence of single loading time on the cyclic ablation of carbide modified C/C composites, a C/C-SiC-ZrC composite was impacted by plasma at 2600 K for 50 s under reciprocating 0.5 (C_0.5_) and 5 s (C_5_), respectively. The composites displayed similar negative mass and rising positive linear ablation rates from C_0.5_ to C_5_. Phases, micro-morphologies, and surface temperature analysis suggested that the partially oxidized SiC-ZrC covering on the ablated sample cracked and was persistently peeled off. The mass gain resulted from the ceramic’s protection of the nearby carbon from complete oxidation. The longer single loading of 5 s caused strengthened thermal chemical reaction and mechanical erosion, which resulted in the bigger linear loss.

## 1. Introduction

C/C-SiC-ZrC composites are one of the most promising materials working in conditions of ultra-high temperatures. Various methods have been developed and successfully prepared the composites, such as chemical vapor infiltration [1], precursor infiltration pyrolysis [2], reactive melt infiltration [3], slurry impregnation [4], sol-gel [5], carbothermal reaction [6], chemical liquid-vapor deposition [7], and so on. Meanwhile, the ablation resistance of the C/C-SiC-ZrC has been greatly studied since the property is vital to its aerospace application. On the one hand, all the constituents (carbon skeleton density [8], substrate carbon species [9], SiC-ZrC content [10], ZrC/SiC ratios [11], nitrogen or rare earth metal oxide doped [12,13]), microstructures (PyC interphase [14], ZrC particles distribution and size [15,16], surface structure unit [17], sandwich-structure [18], intelligent cooling structure, and multi-layer structure [19,20]) and processes (fabrication methods and thermal treatment [21,22]) have been researched continuously to optimize the composite. On the other hand, various tests in diverse environments of ultra-high temperatures such as flame [23], laser [24], and plasma [25] were performed to understand the ablation behavior and mechanism.

Among the large number of investigations, cyclic ablation of modified and coated C/C composites for reused applications has received increasing attention [26,27,28]. Some studies confirmed that cyclic ablation rates were lower than those under single loading when the total impacting time of combustion was the same, because the longer single loading time resulted in higher surface temperatures [29,30,31]. Other studies [32,33] suggested that shorter single loading times resulted in higher cyclic ablation rates for the strengthened thermal shock. Recently, cyclic ablation of C/C-SiC-ZrC-ZrB_2_ under different loading spectra indicated that the ablation rates rose sharply as the single loading time increased from ms to s [34]. The preceding works raised an intriguing issue concerning thermal shock in ultra-high temperature conditions, higher frequency for more times versus fewer cycles of longer single loading, which is more destructive to the C/C-SiC-ZrC composite.

As is well known, both the introduced SiC-ZrC and SiC-ZrC-ZrB_2_ could develop the ablation resistance of C/C composites. However, ZrC played different roles in the oxidation of SiC-ZrC-ZrB_2_ below and above 2000 °C [35,36], and C/C-SiC-ZrC and C/C-SiC-ZrC-ZrB_2_ possessed distinct ablation behaviors [37,38]. Thus, clarifying the cyclic ablation behaviors under different single loading times, especially the not yet reported ms to s, is interesting and helpful to the application of C/C-SiC-ZrC for the nozzle of a repetitive starting engine. The present work mainly compared the cyclic ablation of C/C-SiC-ZrC under 0.5 and 5 s single loading and discussed the conflict of linear and mass ablation rates.

## 2. Experimental Procedure

The C/C-SiC-ZrC composite was prepared by a combination of chemical vapor infiltration (CVI) and precursor infiltration and pyrolysis (PIP). Two-dimensional needle punched carbon fiber felt with a density of 0.45 g/cm^3^ was infiltrated by pyrocarbon to 0.8 g/cm^3^ through CVI using methane gas as a source. Then the porous C/C skeleton was densified by SiC-ZrC through PIP with mixed precursors, and the pyrolysis was performed at 1600–1900 K in a flowing Ar atmosphere. The final density was about 2.2 g/cm^3^ and the SiC/ZrC ratio was designed as 2:3 depending on its outstanding ablation resistance under the oxyacetylene torch [39].

**Figure 1:**
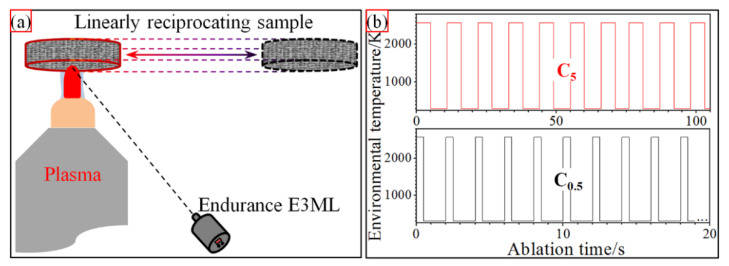
Schematic cyclic ablation (**a**) and loading spectra (**b**).

As shown in Figure 1a, samples with dimensions of Φ30 × 10 mm^3^ were ablated on a self-developed bench. A plasma generator (Multiplaz 3500) provided the jet flow of ionized H_2_O under a working voltage of 160 V and a current of 6 A. The impacted plasma on the sample surface center was about 2600 K. The two test groups were carried out according to the spectra in Figure 1b, respectively. The sample under 10 cycles of 5 s single loading was labeled as C_5_ and that under 100 cycles of 0.5 s was marked as C_0.5_. During ablation, the surface center of the tested sample was monitored by two infrared thermometers of Endurance E3ML and E1RH, which could record the temperature in the range of 323–3473 K with a response time of 20 ms. Finally, ablation rates were obtained through calculation depending on the changes in mass and thickness at the sample center before and after the ablation test. Other details about the preparation and ablation can be found in our previous work [36,39].

The phase, microstructure, morphology, and chemical ingredients of the composites before and after ablation were characterized by X-ray diffraction (XRD-6000 X-ray diffractometer, Shimadzu, Tokyo, Japan) and scanning electron microscopy (SEM, VEGA II XMU scanning electron microscope, TESCAN, Brno, Czech Republic) combined with energy dispersive spectroscopy (EDS, Oxford Instruments, Oxford, UK).

## 3. Results and Discussion

### 3.1. Ablation Characteristics

**Figure 2:**
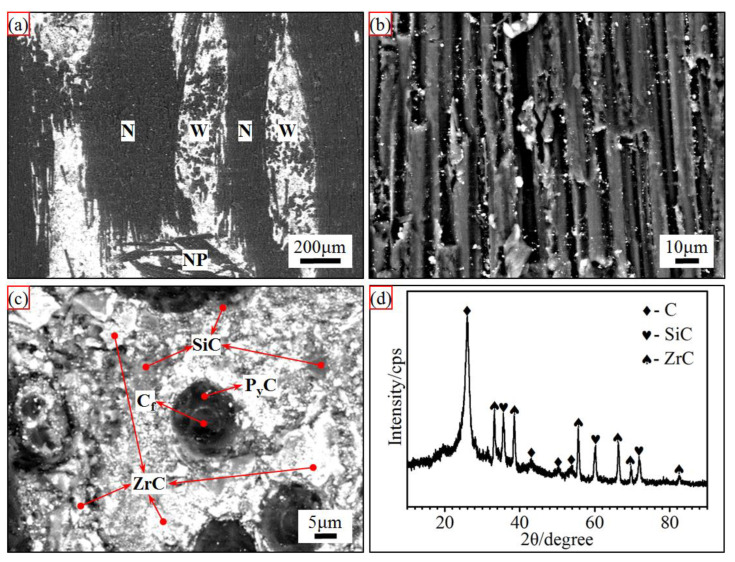
Microstructure and XRD pattern of the prepared C/C-SiC-ZrC composite: (**a**) low magnification; (**b**) non-woven layer; (**c**) web layer; and (**d**) XRD pattern.

Figure 2 shows the microstructure of the prepared C/C-SiC-ZrC composite before ablation. It was clear that the composite was mainly formed by a non-woven layer (marked as N) and a web layer (marked as W) alternately through needle punched fibers (marked as NP). In the N layer, a small amount of introduced ceramic particles are distributed in the fiber bundles, while most of the ceramic is located in the W layer. In addition, the carbon fibers were covered by a thick layer of PyC around 5 μm, and the nearby SiC (the grey phase) and ZrC (the brighter particles) mixed together, which was confirmed by EDS and XRD analysis.

**Figure 3:**
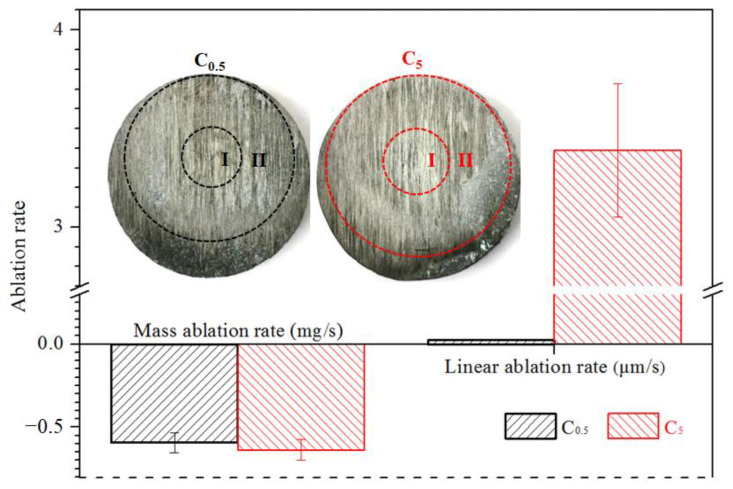
Ablation rates and macro-morphologies of the ablated C/C-SiC-ZrC samples.

After two different ablation processes, the macro-morphologies and ablation rates of the tested C/C-SiC-ZrC samples were shown in Figure 3. From the macro-morphologies, both the two kinds of samples could be divided into two damaged areas: the ablation center region (marked as I), which was a pit in the surface center of the composites; and the surrounding affected region (marked as II), which was relatively flat but the color in this region became lighter than the non-ablated marginal zone. In addition, both the areas of region I and region II under C_0.5_ were smaller than under C_5._ This corresponded to the single loading time.

The ablation rates displayed an interesting conflict as the mass ablation rates were negative whereas the linear ones were positive. As well known, erosion of carbon would lead to decreased mass and volume, while only oxidation of SiC or ZrC might cause negative ablation rates for the composite. In other words, when the mass gain from oxidation of SiC-ZrC exceeded the loss from carbon, the mass ablation rate could be negative; and since only the volume expansion of oxidized SiC-ZrC was bigger than the consumption of composite in combustion, the linear ablation rate could not be positive. Therefore, the relative content and oxidation of the SiC-ZrC ceramics should be responsible for the conflict of ablation rates. This will be covered in greater detail in Section 3.2. When the linear and mass ablation rates were combined, it could be concluded that the ablation under C_5_ was severer than that under C_0.5_. In other words, the longer single loading time (5 s) of plasma was more destructive to the C/C-SiC-ZrC composite than the higher frequency (0.5 s) when the total time was certain. This was similar to our previous study on C/C-SiC-ZrB_2_-ZrC [34], and the C/C-SiC-ZrC presented higher sensitivity to the single loading time in the view of linear ablation rates. However, their mass ablation rates were the opposite. Obviously, the higher ceramic content in C/C-SiC-ZrC should be the key cause.

**Figure 4:**
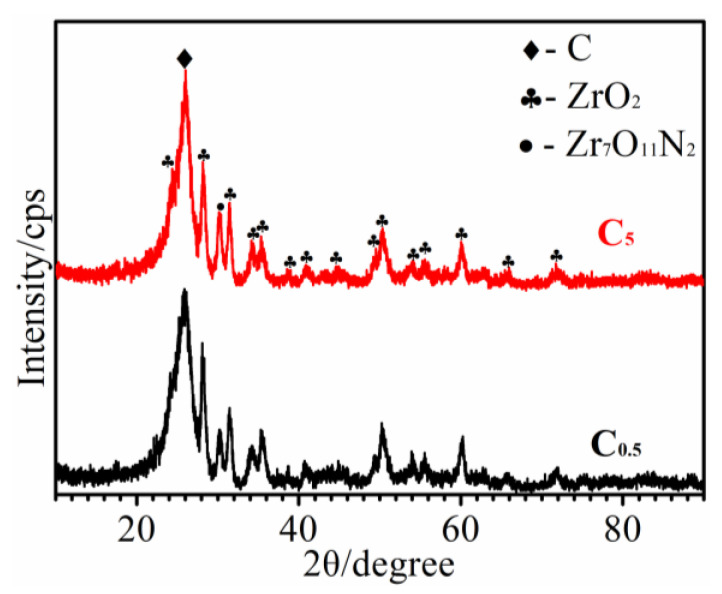
XRD patterns of the ablated C/C-SiC-ZrC under different loading spectrums.

The XRD patterns of C_0.5_ and C_5_ are shown in Figure 4. It could be found that both C_0.5_ and C_5_ only had the oxidation products of ZrC (ZrO_2_ and Zr_7_O_11_N_2_) after ablation, although their surfaces displayed different colors (Figure 3). SiC, ZrC, and SiO_2_ were not detected. Considering the 26 wt% SiC and the rapid unloading of plasma, it could be inferred that the possible SiO_2_ might exist in an amorphous state. Furthermore, the Zr_7_O_11_N_2_ should result from the reaction of ZrC with oxygen in plasma and mixed N_2_ from air.

**Figure 5:**
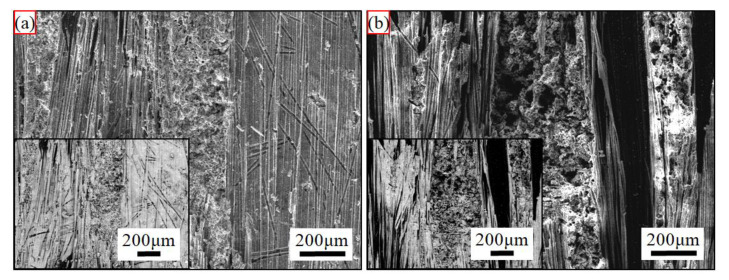
Surface morphologies of the ablated C/C-SiC-ZrC at low magnification: (**a**) under C_0.5_ and (**b**) under C_5_; the insert image at the corner is the corresponding backscattered electron morphology.

**Figure 6:**
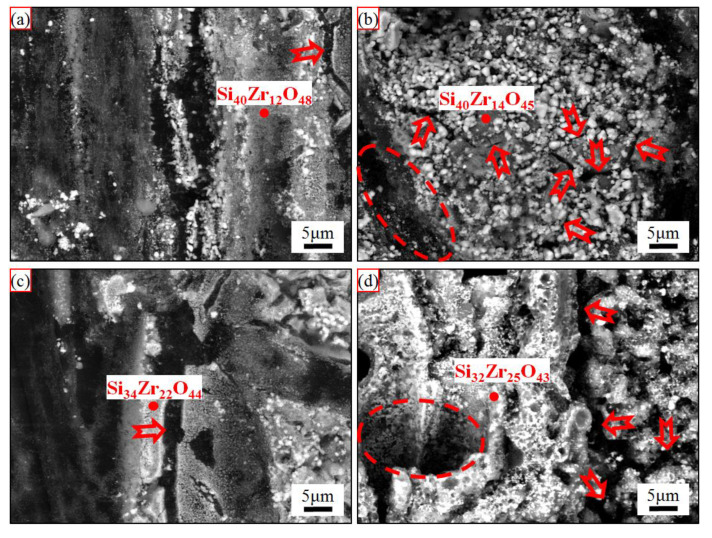
Surface morphologies of the ablated C/C-SiC-ZrC corresponding to the region I in Figure 3 and relevant EDS analysis: (**a**,**b**) under C_0.5_; (**c**,**d**) under C_5_; (**a**,**c**) non-woven layer; and (**b**,**d**) web layer.

The surface central backscattered electron morphologies of the ablated C/C-SiC-ZrC composites under different loading spectra are shown in Figure 5. The ablated surface under C_0.5_ was much flatter than that under C_5_ at this magnification. Additionally, some grooves of oxidized N layer were obvious on the C_5_ surface. In addition, both ablated surfaces of the C/C-SiC-ZrC were covered by white phases, and the covering layer on C_0.5_ was more integrated and even. Further magnifications of the ablated surface centers and relative EDS analysis were presented in Figure 6, which indicated that almost all the original surface was covered by partially oxidized SiC-ZrC. Additionally, all the surfaces cracked. However, the cracks (marked by red arrows) under C_0.5_ were much smaller than those under C_5_. In addition, holes (marked by red ellipse) of oxidized carbon fiber and their surrounding pyrocarbon were clear on the ablated surface under C_5_, whereas some residual carbon (marked by red ellipse) was partially coated by the ceramic particles under C_0.5_. Obviously, oxidation under C_5_ was much severer than that under C_0.5_. Moreover, some of the survived ceramics under C_5_ displayed an overburnt state (Figure 6d), which suggested a higher surface temperature.

**Figure 7:**
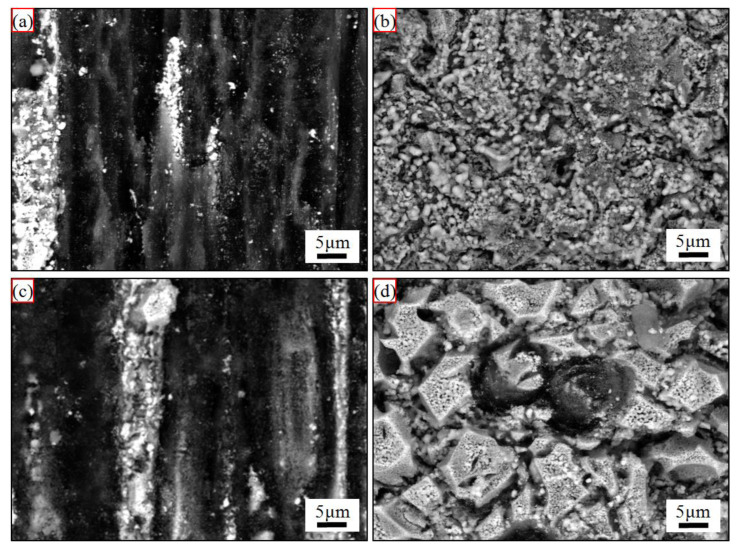
Surface morphologies of the ablated C/C-SiC-ZrC corresponding to the region II in Figure 3: (**a**,**b**) under C_0.5_; (**c**,**d**) under C_5_; (**a**,**c**) non-woven layer; and (**b**,**d**) web layer.

Figure 7 shows backscattered electron morphologies of the regions II (marked in Figure 3) of the ablated C/C-SiC-ZrC composites. No needlelike fiber was found, which suggested that the mechanical erosions under the two spectra were strong. Moreover, the deeper oxidative grooves indicated severer oxidation under C_5_.

**Figure 8:**
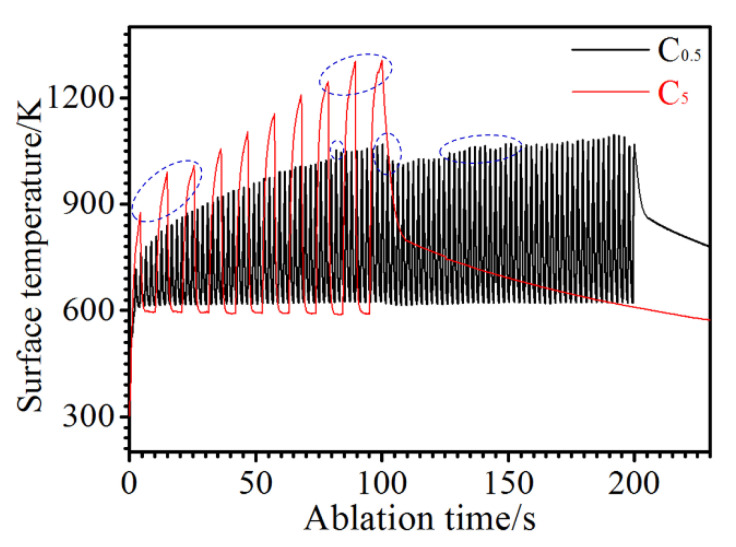
Surface temperatures of the C/C-SiC-ZrC samples during ablation.

Figure 8 shows the surface temperatures of the C/C-SiC-ZrC during ablation. Both surface temperatures rose gradually with the ablation cycles, and the temperature under C_0.5_ was lower than that under C_5_ for its short term of heating. Meanwhile, the surface temperature increased rapidly under each single loading of plasma, which should come from the large temperature difference between the sample and the plasma. In addition, it was obvious that the maximum temperatures under both spectra fluctuated abnormally sometimes (marked by the blue dotted ellipse). The obtained surface temperature was an important signal that reflected the surface states. Thus, it can be deduced that some particles or phases were peeled off during ablation.

### 3.2. Ablation Mechanism

**Figure 9:**
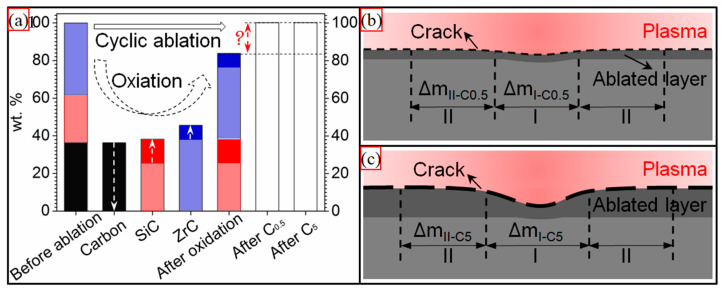
Schematic cyclic ablation mechanism: (**a**) mass percentage of the C/C-SiC-ZrC before and after the cyclic ablation and complete oxidation; (**b**) mass change of different ablated areas under C_0.5_; and (**c**) mass change of different ablated areas under C_5_.

Different from our previous study, where the C/C-SiC-ZrC-ZrB_2_ ablation rates rose sharply from C_0.5_ to C_5_, the C/C-SiC-ZrC had negative similar mass ablation rates. Thus, understanding the conflict between linear and puzzling mass ablation rates of the C/C-SiC-ZrC was essential to clarifying its cyclic ablation mechanism. Based on the relative research, two factors could result in a negative mass ablation rate: formation of a protective layer of Si-Zr-O with proper viscosity [10,40], or considerable mass gain from oxidation of the non-center of the ablated surface [16]. In addition, a porous ZrO_2_ layer from oxidation of ZrC always led to a negative linear ablation rate [13,41,42]. It was clear that only an incomplete partial oxidized SiC-ZrC layer existed (Figure 6 and Figure 7) on the ablated surface and was peeled off persistently under the strong thermal shock of cyclic ablation. Thus, the positive linear ablation rates were reasonable. To better understand the negative mass ablation rates, a schematic mechanism is shown in Figure 9. Assuming the original mass of the C/C-SiC-ZrC was one, the composite could be calculated to be 36.3 wt% C, 25.5 wt% SiC, and 38.2 wt% ZrC based on the ceramic content and ratio described in Section 2. If the composite was oxidized completely, the final mass would be 83.8% of the original value according to the chemical reactions of C, SiC, and ZrC in excess oxygen. However, the actual mass change was positive. Combined with the analysis of Figure 2, Figure 5, Figure 6 and Figure 7, it can be inferred that the partial oxidized SiC and ZrC collapsed and covered the surrounding carbon, which protected the carbon from complete oxidation and could result in a possible mass gain. Under C_5_, the longer single loading brought on higher surface temperature and bigger fluctuations, which caused severer oxidation, collapse, and peeling off. Therefore, more material was eroded in region I. Meanwhile, more residual oxides of SiC-ZrC formed in region II, where thermal shock was much milder than in the center. As a result, more consumption in region I was accompanied by more mass gain in region II. Finally, similar negative mass ablation rates and sharp rising linear ablation rates from C_0.5_ to C_5_ came into being.

## 4. Conclusions

The C/C-SiC-ZrC composite displayed similar negative mass and rising positive linear ablation rates when single loading of cyclic plasma varied from 0.5 to 5 s. The partially oxidized SiC-ZrC coated on the ablated surface, cracked and peeled off, according to phase, micro-morphology, and surface temperature analysis. The mass gain resulted from the ceramic’s oxidation and protection to the nearby carbon. The longer single loading of 5 s caused stronger thermal chemical reaction and mechanical erosion, which resulted in the bigger linear loss.

## Data Availability

The data produced in this study are available from the authors upon reasonable request.
